# Spatial orientation during gondola centrifugation with subjects upright versus supine: Evidence for Gestalt psychological mechanisms in vestibular perception

**DOI:** 10.3233/VES-201527

**Published:** 2021-10-16

**Authors:** Arne Tribukait, Ola Eiken

**Affiliations:** a Department of Clinical Neuroscience, Section for Eye and Vision, Karolinska Institutet, Stockholm, Sweden; b Division of Environmental Physiology, Swedish Aerospace Physiology Center, Royal Institute of Technology, KTH, MTH, School of Chemistry Biotechnology and Health, Solna, Sweden

**Keywords:** Vestibular system, spatial disorientation, self-motion perception, Gestalt psychology, pattern recognition, subjective horizontal, subjective vertical, top-down processing

## Abstract

**BACKGROUND:**

Recent theories suggest that perception of complex self-motion is governed by familiarity of the motion pattern as a whole in 3D.

**OBJECTIVE:**

To explore how familiarity determines the perceived angular displacement with respect to the Earth during a simulated coordinated turn in a gondola centrifuge.

**METHOD:**

The centrifuge was accelerated to 2G (gondola displacement 60°) within 12.5 s. Using visual indicators in darkness, responses to the gondola displacement were recorded with subjects (*n* = 10) in two positions: sitting-upright, facing-forward versus lying-supine, feet-forwards. Each subject underwent 2×2 6-minute runs.

**RESULT:**

When upright, subjects indicated a tilt of initially 18.8±11.3°, declining with *T* = 66±37 s. In the supine position (subject’s yaw plane coinciding with the plane of gondola displacement) the indicated displacement was negligible (–0.3±4.8°).

**CONCLUSION:**

Since the canal system is most responsive to stimuli in yaw, these findings are difficult to explain by bottom-up models. Rather, the motion pattern during acceleration would be recognized as a familiar or meaningful whole (entering a co-ordinated turn) only when the subject is upright. Presumably, the degree of familiarity is reflected in the subject’s ability to discern and estimate a single stimulus component. Findings are discussed in connection with human factors in aviation and the principles of Gestalt psychology.

## 1 Introduction

The present study concerns human perception of complex patterns of self motion, as detected by the organ of balance, and how subjective estimates of a simple stimulus component may depend on the fa-miliarity of the pattern as a whole. Within the fields of vision and audition it has since long been recognized that many complex stimuli, like objects and faces [8] or spoken words, are perceived as integrated wholes with qualities that cannot be the mere products of an algorithm-like system receiving elementary sensory data. Pre-attentive processing levels, or feature detectors [39, 44, 55] are modulated by factors more closely related to the nature of conscious experience. These so-called laws of Gestalt psychology [21] comprise the notions of expectation, familiarity and context. The dichotomy of theoretical approaches to perceptual organization has a counterpart in more ordinary experiences of a contrast between, on the one hand, our ability to distinguish and recognize faces and, on the other hand, the difficulty in recalling the details of a familiar face. Similarly, the acoustic components of a well known word, as formally identified in spectrograms, are notoriously evasive to our conscious mind [12]. Such realizations make the relationships between the outer world, elementary sensory data, and perception a fascinating issue not only to scientists but to artists as well. Thus, several properties of our sensory systems can be highlighted by visual illusions [29], and the structure of music has been called an archetype of the principles for acquisition of sensory information [42].

It might be worthwhile to consider the sense of balance, which is generally regarded as a “silent companion”, against this backdrop. In recent decades there has been an increasing interest in the perception of complex motion patterns. Several mathematical and cybernetic models have been developed in order to explain how component stimuli to the semi-circular canals and otolith organs influence perceived orientation or motion as well as reflexive eye movements. This approach has also led to the identification of stimulus situations where the perceptual outcome cannot be deduced as a function of elementary components, i.e. according to “bottom-up” mechanisms. Psychologically oriented mathematicians have emphasised that self-motion perception may depend on the familiarity of the stimulus pattern as a whole in 3-D [14] and that “top-down” processing and principles of Gestalt psychology should be taken into account also in theories of vestibular perception [15].

A notable example of complex vestibular stimulation, involving rotation in yaw, pitch and roll, is the motion pattern experienced by a running or cycling individual who enters a curve and leans in the direction of the resultant G vector (vectorial sum of the Earth gravity force and the centrifugal force). In aviation, the entering of such a co-ordinated turn is a most fundamental movement pattern. Like runners, pilots strive to maintain alignment between the head-vertical (z) axis and the resultant G vector. Consequently, during co-ordinated turns, the graviceptive systems cannot sense that the aircraft is tilted in roll. If the change in roll attitude is performed rapidly it will, nevertheless, constitute a stimulus to the vertical semicircular canals, similar to that caused by a lateral head tilting in the static 1-g environment [10, 30, 60]. This situation might be considered a conflict between the otolith organs and the semicircular canals. In addition, the runner or pilot will be exposed to an angular-velocity stimulus, the axis of which is gradually changing with respect to the head; in the beginning of the turn this consists mainly in yaw rotation, but if the roll tilt exceeds 45 degrees (i.e. at 1.4G) the pitch-backward component will predominate [13, 23, 47].

**Fig. 1:**
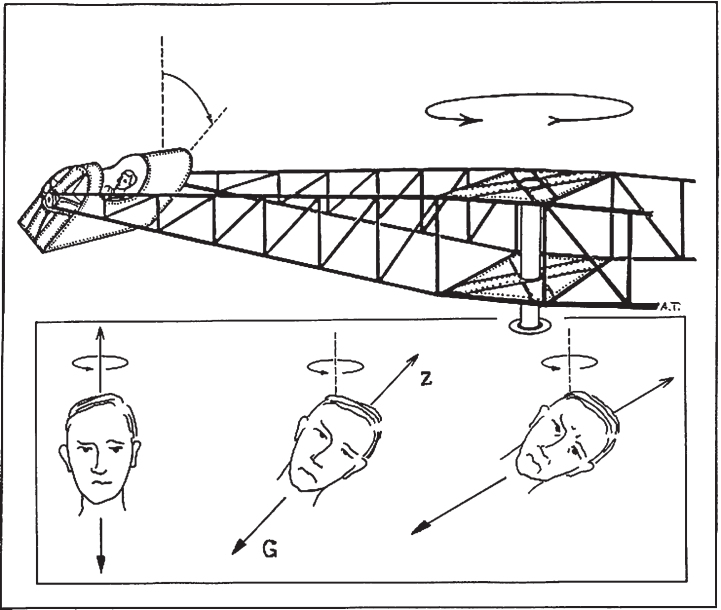
The gondola centrifuge. Acceleration of the centrifuge about its main axle was 7°/s^2^. During acceleration, the cabin is rolled so that the resultant of the Earth gravity force and the centrifugal force remains aligned with the head-to-seat (z) axis of a subject sitting upright in the gondola (except for an inertial component (0.09G) acting posteriorly during acceleration). Thus, the graviceptive systems persistently signal that the head is upright in roll. Nevertheless, the change in roll position is an angular-*displacement* canal stimulus (which after 12.5 s of acceleration amounts to 60° as the resultant gravitoinertial force vector reaches the plateau value 2G). In addition, because of the change in roll position, the angular-*velocity* stimulus (which is the time integral of the angular acceleration of the centrifuge), related to the rotation of the centrifuge about its main axle, gradually changes from yaw-left to near pitch backward.

As pointed out by McGrath and co-workers [13, 23], a similar stimulus situation can be created by means of a large centrifuge with tangentially pivoted gondola (Fig. 1). A subject seated facing forwards in the gondola will be exposed to a gravitoinertial force vector that is persistently acting in parallel with the head-vertical axis (except for the tangentially acting inertial component during acceleration of the centrifuge). The swing out of the gondola during acceleration of the centrifuge is a roll-plane angular-displacement stimulus, equivalent with the banking of an airplane during the entering of a turn. Nevertheless, since the centrifuge has a much smaller radius than the trajectory of a turning aircraft, the angular-velocity stimulus will, for a given change in roll attitude, be much greater in the centrifuge [54].

An estimate of the perceived roll tilt can be obtained by means of an adjustable luminous line in otherwise complete darkness. The subject’s task is to adjust the line so that it is perceived as horizontal (or vertical); the deviation from the true horizontal is recorded in degrees. This measure of spatial orientation is denoted the subjective visual horizontal (SVH) [6] or vertical (SVV) [27]. Although visual measures of spatial orientation are not always equivalent with measures obtained by means of postural or somatosensory methods [5, 28], the sensitivity of the SVH to vestibular stimuli makes it valuable in studies on canal-otolith interaction or the effects of complex canal stimuli. In test subjects, seated upright facing forwards in the gondola, acceleration of the centrifuge from stationary to a pre-determined G level causes a sensation of being tilted towards the centre of the centrifuge. This is reflected in a tilt of the SVH with respect to the inter-aural axis of the subject. In non-pilots, the initial SVH tilt is, on average, approximately 30 per cent of the real roll tilt and it usually declines with a time constant of 1-2 minutes, reflecting the decaying memory for canal information on angular *displacements* [48, 51].

Conspicuously, when subjects were seated facing backwards in the gondola, the SVH tilt induced by acceleration of the centrifuge was substantially smaller [47]. Therefore, the magnitude of perceived roll tilt cannot be dependent solely on the roll (an-gular-*displacement*) component of the canal stimulus, since this is of equal magnitude (but of opposite sign) when the subject is in the backward position. One possible explanation is that the *transition* from yaw to near pitch *backward* angular velocity during acceleration facing forwards is familiar to the subject, thus supplementing or confirming the roll angular-displacement stimulus. A contrasting possibility is that the angular-velocity stimulus during acceleration facing backwards (i.e. transition from yaw to pitch-*forward* angular velocity) is *unfamiliar* and thereby interferes with the subject’s ability to discern the roll-plane component.

To extend this reasoning, the pattern of canal stimuli in yaw, roll and pitch during acceleration of the centrifuge would be most unfamiliar when the subject is lying supine, feet forwards, in the gondola. On the other hand, this entails that the subject’s yaw plane coincides with the swing-out of the gondola. As considered in the discussion section, several lines of evidence show that humans are more apt to perceive canal stimuli in yaw than in roll and pitch; in addition, there are reasons to believe (as discussed in section 4.2) that if a test subject is positioned supine in the gondola, then the persistent otolithic signal acting along the subject’s naso-occipital axis would not interfere with (or counteract) the canal message for yaw angular displacement during acceleration of the centrifuge. If the perceived angular displacement is nevertheless of smaller magnitude when subjects are in the supine position this would support the notion that the brain identifies certain complex vestibular stimuli as meaningful wholes, one case being the entering of a co-ordinated turn.

Consequently, the aim of the present study was to establish whether the angular displacement of the gondola during acceleration of the centrifuge is underestimated to a similar degree when the subject is in the supine position (feet forwards) as when he or she is sitting upright. For the supine position there seem to be two contrary possibilities: (i) the brain could be capable to single out the yaw-plane component, estimating its magnitude independently of the other stimulus components; (ii) if the stimulus pattern as a whole is unfamiliar, this could prevent the perception of the yaw-plane component; any response to the latter would be meaningless in a context that cannot be recognized.

## 2 Methods

### 2.1 Subjects

Ten healthy males, aged 25–52 (mean = 34, me-dian = 33) years, were recruited to the study. As regards experience of coordinated turns, the subjects were not motorcycle drivers and they did not have any experience of manoeuvring an aircraft. All had earlier participated in centrifuge experiments. The subjects had all had an overview of the centrifuge; they were aware of its size, the direction of rotation and knew that the gondola was always hanging in the direction of the resultant gravitoinertial force vector. When installed in the gondola and instructed about the tasks, they could also observe the equipment, and check how to adjust the visual target by means of the push buttons on the remote control.

### 2.2 Study design

**Fig. 2:**
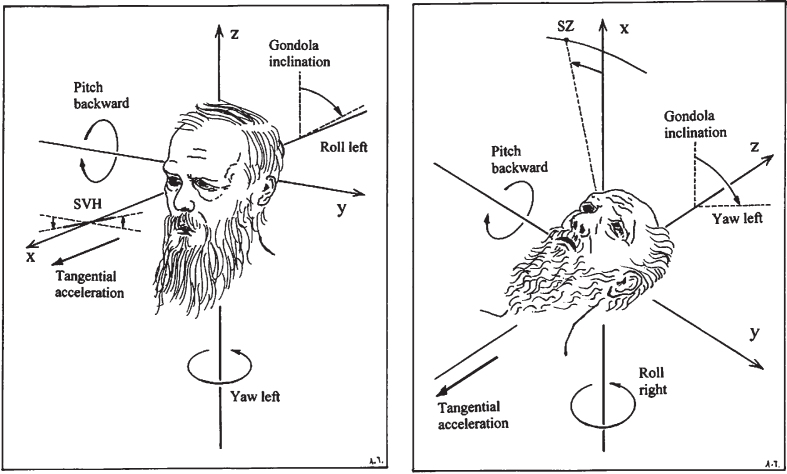
Stimulus conditions during acceleration of the centrifuge and principles for measurement of the SVH and SZ. When the subject is upright, the gondola inclination is a roll-left angular displacement stimulus. When the subject is supine, feet forwards, the gondola inclination is a yaw-left displacement. If the subject perceives the angular displacement of the gondola, then, this will be reflected in a deviation of the SVH or SZ in the opposite direction.

The subjects participated in two experimental sessions (A and B), separated by an interval of 1-2 days (for subjects No. 9 and 10, the interval was 6 hours). Each session comprised two centrifuge runs. In session A, measurements of the SVH was performed with the subject in the upright position; in session B, the subjective zenith (SZ) was measured with the subject in the supine position (Fig. 2). The order of the sessions was balanced between the subjects.

### 2.3 Equipment and general procedures

The experiments were performed in the swing-out gondola centrifuge at KTH in Solna, Sweden. The radius of this centrifuge is 7.25 metres and its rotation is anti-clockwise (as seen from above). The tangentially pivoted gondola deflects in the direction of the resultant force vector (vectorial sum of the Earth gravity force and the centrifugal force) (see Fig. 1). The gondola was equipped with video surveillance and the test subject could always communicate with the experimenter via a two-way intercom system. The subject’s heart rate and rhythm were monitored continuously by means of electrocardiography.

The centrifuge was accelerated from stationary to 2G. At 2G the angular velocity of the centrifuge about its main axle is 88°/s and the inclination (swing out) of the gondola is 60°. With an angular acceleration of the centrifuge about its main axle of 7°/s^2^ the 2G level was attained within 12.5 s. Thus, the mean angular velocity of the swing-out of the gondola was well above the stimulus threshold for the semicircular canals. The recording time at 2G was 6 minutes. In the pauses between runs the gondola was opened and the light was turned on for 5 min.

### 2.4 Stimulus characteristics

**Fig. 3:**
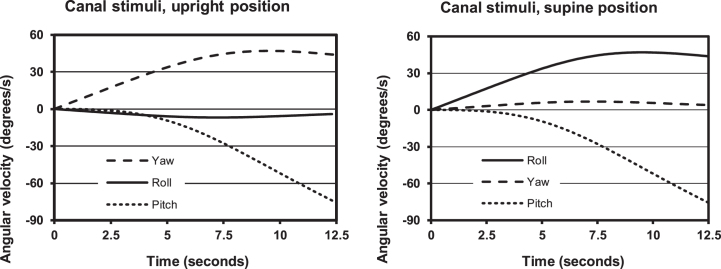
Angular velocity stimulus for the three head-fixed planes, i.e. roll (about the x axis), pitch (about the y axis) and yaw (about the z axis). Left diagram: subject upright; right diagram: subject supine. For a given point in time, the angular velocity components in yaw and pitch (subject upright) or roll and pitch (subject supine) are determined by the angular velocity of the centrifuge about its main axle and the inclination of the gondola.

As suggested in Fig. 1, the resultant gravitoinertial force vector does not change direction with respect to the subject, except for the tangential inertial component (0.09 G) during acceleration of the centrifuge. However, because of the angular displacement (swing out) of the gondola during acceleration of the centrifuge the orientation of the subject gradually changes with respect to the plane of centrifuge rotation. The pattern of canal stimuli can be characterized as the yaw, pitch and roll angular velocity components depicted in Fig. 3. The logical relationships between these components are perhaps more easily imagined if also considering Table 1. Although the physical cause of semicircular canal stimulation (i.e. endolymph displacement and cupula deflection) is angular *acceleration*, when it comes to perceptual or oculomotor responses it is more expedient to consider stimuli and responses in terms of angular *velocity* (as measured in degrees/second) or angular *displacement* (measured in degrees). This way of characterizing canal stimuli is appropriate on the presumption that angular accelerations and stimulus durations are not far beyond the ranges of natural behaviour. To a subject seated upright in the gondola, the roll-left angular *displacement* of the gondola (amounting to 60° at 2G) also entails a *transition* from yaw-left to pitch backward angular *velocity*. When the subject is supine, the yaw-left angular displacement is accompanied by a transition from roll-right to pitch backward angular velocity. These rather complex patterns follow from the 7°/s^2^ acceleration of the centrifuge about its main axle and the concomitant swing out of the gondola.

**Table 1:** Stimulus conditions during acceleration of the centrifuge characterized in terms of angular displacement (the swing out of the gondola, measured in degrees), angular velocity components (measured in degrees/s), forming a specific pattern because of the swing out of the gondola with respect to the plane of centrifuge rotation, and the direction of the resultant G vector. In addition, due to the tangential acceleration of the gondola there is a gravitoinertial component (0.09G), directed backwards (subject upright) or headwards (subject supine)

Condition	Angular *displacement*	Angular *velocity* transition	Gravitoinertial force vector
Seated upright facing forwards	Roll left	Yaw left to pitch backward	Along the head-to-seat (z) axis
Lying supine feet forwards	Yaw left	Roll right to pitch backward	Along the naso-occipital (x) axis

#### 2.5.1 Experimental session A, SVH in the upright position

In session A, the subject was seated upright, facing forwards in the gondola and fixed by means of safety belts and a head holder. The head was positioned so that a line from the external auditory meatus to the inferior margin of the orbit was tilted upwards (nose up) approximately 10 degrees with respect to the gravitational horizontal. The inter-ocular line was gravitationally horizontal.

In front of the subject there was a line of red luminous diodes, subtending a visual angle of 6.5°. Connected to a low-voltage DC motor and a digital angular encoder (Heidenhain ERN 1080), the line could be rotated about the subject’s visual (naso-occipital) axis (i.e. in the fronto-parallel plane).

Every time the line was switched on the subject adjusted it, using two push-buttons on a remote control so that it appeared to be horizontal (i.e. coincided with the subject’s spontaneous imagination of the horizon of the external world). Thus, in case of any sensation of being tilted, the subject should indicate the horizon in relation to which he felt tilted, not the transversal plane of the head. When pleased with a setting, the subject pressed a third button, which extinguished the line. The deviation of the line from the gravitoinertial horizontal was recorded with an accuracy of 0.1°. Before it was switched on again the line was rotated 5–20° (randomly), alternately clockwise and counter-clockwise with respect to the subject’s latest setting. Except for the line the gondola was completely darkened. Prior to the first centrifuge run a series of 8 settings of the luminous line was obtained; prior to the second run 4 settings were made. During centrifugation, data collection commenced as soon as the 2-G level was attained (*t* = 0). As a rule, subjects made 3–5 settings per minute. A positive value of the SVH denotes a response in the direction compensatory to the inclination of the gondola.

#### 2.5.2 Experimental session B, SZ in the supine position

The subject was in the supine position with the feet pointing in the direction of tangential motion. He was fixed by means of a harness and a head holder (a padded groove with a strap around the forehead).

Above the head of the subject (at a distance of 0.8 m from the subject’s eyes) there was a black screen on which a red laser dot could be projected. The laser pointer was mounted rostrally to the subject’s head on an axle parallel with his longitudinal (z) axis and intersecting the inter-ocular axis in the midline. The pointer had a caudal inclination compensating for its distance in the z direction from the interocular line. The axle was connected to a low-voltage DC motor and a digital angular encoder (Heidenhain ERN 1080). Thus, the position of the dot could be adjusted strictly in the transverse (right-left) direction in a plane perpendicular to the subject’s z axis and intersecting the eyes. The screen was curved so that the distance between the dot and the root of the subject’s nose was always the same.

The subject was instructed to adjust the dot so that it appeared to be *gravitationally* above the root of the nose. Thus, in case of any sensation of being tilted in yaw, the subject should set the dot in a position from where a falling drop of water would hit the face between the eyes; he should *not* indicate his own median plane. Every time the dot appeared, the subject adjusted it, using two push-buttons on a remote control. When pleased with a setting, the subject pressed a third button, which extinguished the dot. The deviation from the gravitoinertial zenith was recorded with an accuracy of 0.1°. Before it was switched on again the laser pointer was displaced 5–20° (randomly), alternately to the left and right with respect to the subject’s latest setting. Except for the dot the gondola was completely darkened.

In order to make the subject acquainted with the task, and to ascertain that he would not confuse the *allocentric* task of indicating the SZ with the *egocentric* task of setting the luminous dot in the perceived median plane of the head (subjective straight ahead), prior to each centrifuge run the subject performed a series of 8 settings of the dot in the supine-neutral position. Thereafter 8 settings were made also during static tilts of 10 degrees to the right and left about the Earth-horizontal z (head and body long) axis; intervals in the neutral position between these tilts were approximately 2 minutes. Prior to the second run, the order of tilts was reversed. During centrifugation, data collection commenced as soon as the 2-G level was attained (*t* = 0). As a rule, subjects made 3–5 settings per minute. According to the sign convention, a response that is compensatory to the gondola inclination during centrifugation is negative.

### 2.6 Definitions and treatment of data

**Fig. 4:**
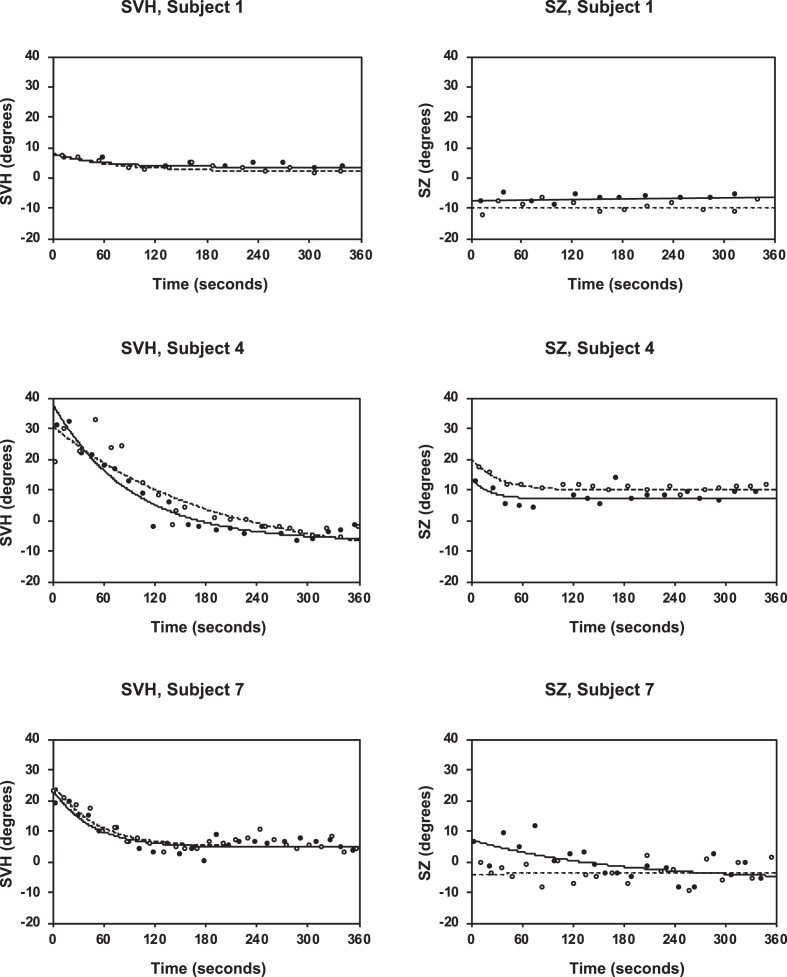
Changes with time of the SVH (left diagrams) and SZ (right diagrams) after acceleration from stationary to 2G. Each data point represents one setting of the luminous line (SVH) or luminous dot (SZ). The lines represent best-fitting exponential functions. Every subject underwent two centrifuge runs in each condition (run 1: black symbols, continuous lines; run 2: open symbols, dotted lines). These three subjects represent the extremes (subject 1 and 4) and average (subject 7) of deviations in the SVH.

For each subject the mean of the settings made at 1g prior to each run was calculated. Thus, for the individual there were two 1-g values for the SVH and two for the SZ. According to the conventions depicted in Fig. 2, clockwise deviations of the SVH (from the subject’s point of view), as well as deviations of the SZ to the left of the midline, are denoted positive. Thus, a true compensatory response to the swing out of the gondola will have a positive sign for the SVH but a negative for the SZ. For each centrifuge run, the function SVH (or SZ) = Ae^-t/T^+C was adapted (least square fit) to the data points (see Fig. 4). Time 0 is defined as the beginning of the 2-G plateau. The constant A represents the initial deviation (response) with respect to an asymptote C. This third parameter (C) is motivated by the fact that normal individuals, while gravitationally upright in the static 1-g environment, often have a deviation (albeit usually < 2.5°) from the true gravitational horizontal [6, 46]; at an increased gravitoinertial force vector, acting in parallel with the subject’s head-to-seat (z) axis, this static component of the SVH can be considerably greater [51]. T is the time constant for exponential decay, t is time. Least squares curve fitting was performed by means of Microsoft Excel Problem Solver, using the so-called multistart for global optimization. Statistical comparisons of group means were made using paired *t*-tests or one-way ANOVA with repeated measures. Linear regressions were also used with data arranged pairwise.

### 2.7 Ethical considerations

The subjects participated with their written in-formed consent and were free to withdraw at any time during the study. The test procedures were in accordance with the declaration of Helsinki and were approved by the human ethics committee in Stockholm.

## 3 Results

### 3.1 SVH in the upright position

In the 1-g environment the SVH was close to the true gravitational horizontal (mean±1SD): –0.86±1.50° (prior to run 1) and –0.55±1.66° (prior to run 2). There was a correlation between values obtained prior to run 2 and those obtained prior to run 1 (linear regression: *r* = 0.69, *p* = 0.03, *n* = 10).

Results of fitting the exponential function SVH =Ae^-t/T^ + C to data obtained at the 2-G plateau are summarized in Table 2a. Examples are shown in Fig. 4. The constant A was: 19.9±11.8° (run 1) and 17.6±11.2° (run 2); there was a correlation between data from run 1 and those obtained at run 2 (linear regression: *r* = 0.92, *p* = 0.0002, *n* = 10). For each individual the average for the two runs was calculated, and then the group mean: 18.8±11.3°.

**Table 2a:** SVH at 1g prior to centrifugation and results of fitting the function SVH = Ae^-t/T^ + C to data obtained during centrifugation. R1, run 1; R2, run 2; M, mean for R1 and R2

Subject	SVH at 1g (degrees)			A (degrees)			C (degrees)			T (seconds)		
	R1	R2	M	R1	R2	M	R1	R2	M	R1	R2	M
1	1.0	1.0	1.0	4.5	5.9	5.2	3.5	1.9	2.7	54	79	67
2	–0.9	–0.1	–0.5	29.3	16.0	22.6	0.6	4.6	2.6	83	39	61
3	0.8	1.1	1.0	16.7	15.1	15.9	–2.5	3.2	0.3	196	50	123
4	–1.5	–2.6	–2.0	45.1	44.1	44.6	–6.8	–13.3	–10.1	90	189	139
5	–1.4	0.8	–0.3	8.0	10.2	9.1	–0.7	4.0	1.6	56	20	38
6	–3.8	–1.8	–2.8	17.7	15.6	16.7	–4.6	–3.2	–3.9	64	65	64
7	0.7	0.6	0.7	19.0	21.0	20.0	4.6	4.8	4.7	45	49	47
8	–0.2	0.7	0.3	10.0	5.0	7.5	1.5	3.1	2.3	53	29	41
9	–2.2	–2.5	–2.4	24.5	24.8	24.6	–3.8	–2.0	–2.9	50	65	57
10	–1.1	–2.8	–2.0	24.3	18.5	21.4	–1.7	–2.5	–2.1	12	33	23
Mean	**–0.9**	**–0.6**	**–0.7**	**19.9**	**17.6**	**18.8**	**–1.0**	**0.1**	**–0.5**	**70**	**62**	**66**
1 SD	**1.5**	**1.7**	**1.4**	**11.8**	**11.2**	**11.3**	**3.6**	**5.6**	**4.4**	**49**	**48**	**37**

The constant C was: –0.98±3.63° (run 1) and 0.06±5.58° (run 2); there was a correlation between data from run 1 and run 2 (linear regression: *r* = 0.79, *p* = 0.0066, *n* = 10). The mean for run 1 and 2 was: –0.46±4.37°. There was a correlation between C and the SVH at 1g (*r* = 0.85, *p* = 0.002, *n* = 10).

The time constant was: 70.3±49.0 s (run 1) and 61.7±48.2 s (run 2). There was no correlation be-tween data from the two runs (linear regression: *r* =0.17, *p* = 0.65, *n* = 10).

### 3.2 SZ in the supine position

**Fig. 5:**
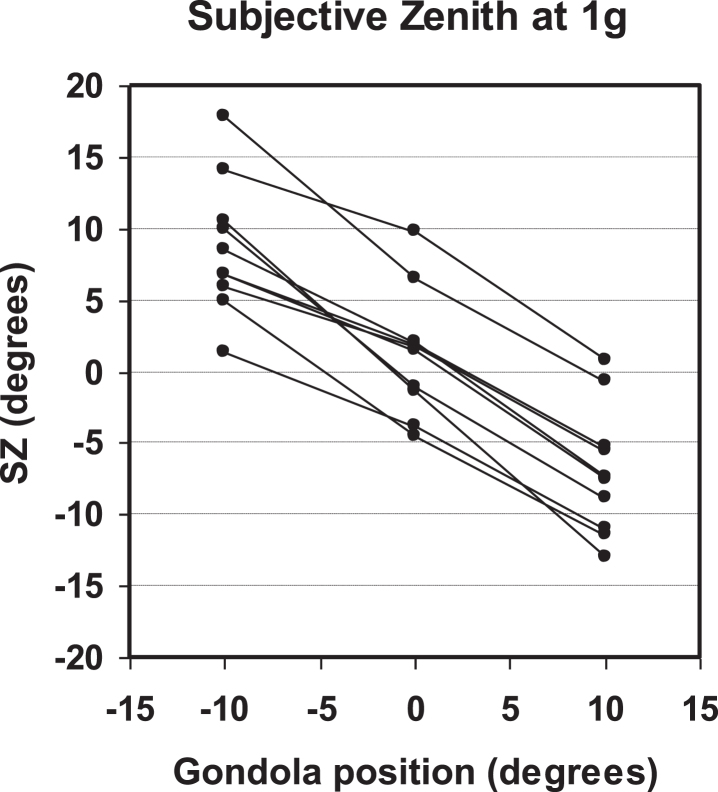
Perception of static tilt about an Earth-horizontal yaw axis. Each data point represents the mean of 16 settings of the luminous dot in one individual. For each individual the data points for the three positions are connected by lines. There was a substantial inter-individual variability in the neutral position. However, the response to static tilt was independent of this baseline value. See also Table 3.

At 1g also the SZ was, on average, close to the true zenith (means±1 SD); 0.86±4.16° (prior to run 1), 1.72±4.98° (prior to run 2), 1.29±4.39° (overall mean). There was a correlation between values obtained prior to run 2 and those obtained prior to run 1 (linear regression: *r* = 0.85, *p* = 0.0020). All subjects responded consistently to static tilts of the gondola by displacing the SZ in the compensatory direction. Individual means for the neutral and tilted positions are presented in Table 3 and Fig. 5. A *relative* measure (gain value) of an individual’s ability to respond to the static tilts (10° to the right and left) can be obtained as the ratio between, on the one hand, the difference in SZ between the tilted positions and, on the other hand, the corresponding difference in gondola position: (SZ_right_ - SZ_left_)/(20°). Individual gain values are presented in Table 3. The group mean for the gain was 0.78±0.19.

As to the sensation of the gondola’s angular displacement during acceleration of the centrifuge, all subjects verbally confirmed that there was a great difference between the two body positions; whereas they experienced a tangible roll-left angular displacement when they were sitting upright, in the supine position there was no sensation of yaw-plane angular displacement. Rather, there were reports on a sensation of being rotated or tilted in pitch, with the feet elevated relative to the head. The combination of perceived feet-forward acceleration, pitch-backward rotation and the absence of any sensation of yaw angular displacement could even generate, during the first seconds of centrifuge acceleration, a perception of self motion along a trajectory that was initially horizontal but soon curved upwards so that the subject felt as if being accelerated vertically with the feet pointing towards the sky. When the sensation of pitch rotation had declined (within a few seconds) subjects felt as if lying stationary in the neutral supine position.

Results of exponential curve fitting to SZ data are summarized in Table 2b. In general, the change during the 6 min recording period was small in comparison with the RMS error. For subject 5, curve fitting to data obtained during run 1 resulted in unreasonable values for the asymptote C and the time constant, as if he would in due course experience an angular displacement exceeding 360 degrees. Since C is, in principle, essential for obtaining an adequate estimate of A (A is a measure of the canal effect we are interested in, whereas C represents a static component), for subject 5, curve fitting to data for run 1 was re-done with the asymptote C pre-set to his preceding 1 g value. This procedure can be justified by results from the other 9 subjects. Namely, the difference between 1 g values (1.29±4.39°, *n* = 9, mean of values obtained prior to run 1 and 2) and values of C (1.09±4.46°, *n* = 9, mean for run 1 and 2) was negligible. Further, there was a significant correlation between 1 g values and C (linear regression, *r* = 0.81, *p* = 0.009, *n* = 9).

**Table 2b:** SZ at 1g prior to centrifugation and results of fitting the function SZ = Ae^-t/T^ + C to data obtained during centrifugation. Please, note that a true response (A) to the gondola displacement has a negative sign. Time constants were not considered reliable in cases with very small responses (A) or if the value for T exceeded the recording interval (360 s) by a factor 4 or more; in such cases T is not shown. Notably, values for the asymptote C were similar to 1 g values. For subject 5, curve fitting to data for run 1 was done with C pre-set to the 1 g value (see main text). Values in parentheses are not included in group means. R1, run 1; R2, run 2; M, mean for R1 and R2. Individual 1-g values under R1 and R2 are means of 8 settings

Subject	SZ at 1g (degrees)			A (degrees)			C (degrees)			T (seconds)	
	R1	R2	M	R1	R2	M	R1	R2	M	R1	R2
1	–1.2	–6.4	–3.8	–4.0	–5.2	–4.6	–3.5	–4.6	–4.1
2	–0.7	–1.4	–1.1	–2.8	0.9	–1.0	0.0	–2.0	–1.0
3	9.7	10.0	9.8	5.2	0.0	2.6	5.3	8.6	7.0	400
4	4.5	8.7	6.6	10.5	6.9	8.7	9.9	7.0	8.5	29	20
5	2.0	1.8	1.9	–8.2	0.0	–4.1	(2.0)	–0.9	(0.5)	238
6	–5.9	–3.0	–4.5	–5.5	–5.7	–5.6	–0.6	3.5	1.4	667	588
7	–2.4	–0.4	–1.4	13.4	–0.6	6.4	–6.3	–3.4	–4.9	169
8	1.2	1.8	1.5	2.0	3.6	2.8	3.6	0.0	1.8	278
9	1.1	2.9	2.0	9.0	–12.5	–1.7	–2.1	5.5	1.7	34
10	0.3	3.2	1.8	–0.7	–0.1	–0.4	0.0	–1.2	–0.6
Mean	**0.9**	**1.7**	**1.3**	**1.9**	**–1.3**	**0.3**	**0.7**	**1.2**	**1.1**
1 SD	**4.2**	**5.0**	**4.4**	**7.4**	**5.4**	**4.8**	**4.9**	**4.6**	**4.5**

**Table 3:** The SZ at 1g. Prior to each centrifuge run (1st and 2nd), each subject made 8 settings of the luminous dot in the supine neutral position and at 10° of static tilt to the right and left. Thus, each individual value in columns denoted 1st and 2nd is the mean of 8 settings. M is the overall mean. The gain value for the SZ responses to static tilt was obtained as the ratio between the difference between values for tilt to the right and left (M_right_ minus M_left_) divided by the difference in gondola position (20°)

Subject	Neutral			10° Right			10° Left			Gain
	1^st^	2^nd^	M	1^st^	2^nd^	M	1^st^	2^nd^	M
1	–1.2°	–6.4°	–3.8°	2.8°	0.0°	1.4°	–7.5°	–14.3°	–10.9°	0.62
2	–0.7°	–1.4°	–1.1°	9.6°	10.3°	10.0°	–10.5°	–7.2°	–8.9°	0.94
3	9.7°	10.0°	9.8°	15.8°	12.5°	14.1°	–2.4°	4.1°	0.8°	0.66
4	4.5°	8.7°	6.6°	19.9°	15.7°	17.8°	–2.5°	1.1°	–0.7°	0.92
5	2.0°	1.8°	1.9°	6.7°	6.8°	6.8°	–3.5°	–7.0°	–5.2°	0.60
6	–5.9°	–3.0°	–4.5°	4.6°	5.3°	4.9°	–13.0°	–9.9°	–11.4°	0.82
7	–2.4°	–0.4°	–1.4°	9.6°	11.6°	10.6°	–11.3°	–14.8°	–13.0°	1.18
8	1.2°	1.8°	1.5°	8.4°	5.2°	6.81°	–6.6°	–8.4°	–7.5°	0.72
9	1.1°	2.9°	2.0°	4.3°	12.6°	8.5°	–7.2°	–7.4°	–7.3°	0.79
10	0.3°	3.2°	1.8°	5.4°	6.4°	5.9°	–5.4°	–5.5°	–5.5°	0.57
Mean	**0.9**°	**1.7**°	**1.3**°	**8.7**°	**8.6**°	**8.7**°	**–7.0**°	**–6.9**°	**–7.0**°	**0.78**
1 SD	**4.2**°	**5.0**°	**4.4**°	**5.4**°	**4.7**°	**4.7**°	**3.7**°	**5.9**°	**4.5**°	**0.19**

The constant A was 1.89±7.38° (run 1) and –1.27±5.39° (run 2). There was no correlation between values from run 1 and 2 (*r* = 0.09, *p* = 0.80).

The constant C was: 0.70±4.91° (run 1, *n* = 9) and 1.24±4.59° (run 2, *n* = 10). There was a correlation between data from run 1 and run 2 (linear regression: *r* = 0.69, *p* = 0.039, *n* = 9). The mean for run 1 and 2 was: 1.09±4.46° (*n* = 9). Since most subjects displayed very small changes in SZ during the recording period, and because the variability in the settings were considerable, values for the time constant cannot be considered as physiologically informative. Also, a value of T exceeding the recording period by a factor 5–10 would not be reliable. Thus, although in some cases the SZ seemed to follow an exponential decay function, the present data do not permit any statistical analysis of the time constant at group level.

### 3.3 SZ in relation to SVH

The constant A was considerably larger for SVH (18.8±11.3°; mean for run 1 and 2) than for SZ (0.31±4.76; mean for run 1 and 2). Figure 4 shows the SVH and SZ data for three subjects. Notably, the subject who had the greatest SVH responses (Subject 4) showed responses with approximately exponential decline also with the SZ; those responses were, however, not in the compensatory direction. In order to enable comparisons between the magnitude of the SVH and SZ responses, for each individual the sign of the SZ value was first changed; thus, for both the SVH and SZ a true response to the gondola tilt during centrifugation is represented by a positive sign (in contrast to the values shown in Table 2b and Table 3, which are in accordance with the sign convention). A One-Way ANOVA with repeated measures shows that the difference between conditions was highly significant (*p* < 0.0001), whereas there were no differences between run 1 and run 2 (see Tables 2a and 2b). The constant A for SVH tended to correlate with A for SZ (linear regression: *r* = 0.61, *p* = 0.06). However, if subject 4, who had the largest deviations in both conditions, is not included there is no correlation (*r* = 0.23, *p* = 0.55, *n* = 9).

## 4 Discussion

### 4.1 Essentials of the findings

The present findings unequivocally show that visual measures of perceived gondola inclination during centrifugation differ considerably between the upright and supine positions. When the subject is seated upright, the 60-degree angular displacement (swing out) of the gondola during acceleration of the centrifuge is a roll-plane canal stimulus. The resulting SVH tilt was on average 19 degrees. To a subject in the supine position the swing out of the gondola is a yaw-plane stimulus. Deviations of the SZ were close to zero. The present measurements of the SZ at 1g reveal that the subjects, while in the supine position, are able to perceive and indicate tilts about the z axis with a stimulus-response relationship (gain) of 0.78±0.19.

As to the comparatively large inter-individual variability in the 1-g values for SZ in the supine-neutral position, one possible explanation is that slight asymmetries in the vestibulo-ocular system, which are likely to be compensated during upright locomotion, may become manifest in the supine position, where humans rarely have visual references that would lead to correction of misperceptions of the subjective zenith. Thus, for comparison, in normal subjects, seated upright, the visually indicated subjective straight ahead (SSA) is more accurate, rarely deviating more than 3.5° from the true median plane [18]. Another factor is differences in relative eye dominance, which is known to vary considerably between individuals and determines the baseline projection centre for perceived visual directions [16]. Notably, since perceived body orientation is governed to high extent by gravity receptors in the abdomen, whereas visual measures of spatial orientation are mainly determined by the vestibular receptors [28], a deviation of the SZ from the true gravitational vertical should not be interpreted as due to a sensation of body tilt.

The gain value (0.78) for the SZ response to static yaw tilt is notably smaller than the 1.0 gain reported for static tilts of 10–30° in roll [46] and pitch [50]. An explanation that lies near at hand is that when the subject is supine, the otolith organs are not, according to the shear-force principle, optimally oriented for detecting moderate changes in head orientation. Nevertheless, a basic question is whether this gain could rather represent a tendency to under-report, using a visual pointer, perceived angular displacements in yaw (whether induced by static tilt or canal stimulation). Even if this were the case, the gain suggests that the ability to respond to perceived changes in yaw position was sufficient for the present comparison between the SVH and SZ responses during centrifugation. Further, at the individual level, there was no tendency for subjects with smaller gains during static tilt to have smaller responses also during centrifugation; a linear regression analysis rather suggests an opposite tendency (*r* = 0.55, *p* = 0.10). Also the inter-individual variability combined with the reproducibility of measurement variables, and of parameters obtained via curve fitting, indicates that the lack of SZ responses during centrifugation is not due to an inherent flaw in the procedure. Finally, all subjects denied having felt any yaw tilt during centrifugation.

The present findings obtained during centrifugation complement those of earlier experiments where the perception of roll tilt was recorded with subjects facing forwards and backwards in a swing-out gondola centrifuge; deviations of the SVH were considerably smaller when subjects were in the backward position [47]. Also, measurement of the visually perceived eye level with subjects in *transverse* positions (facing the centre or periphery of the centrifuge) has yielded measures of perceived (pitch) angular displacement notably smaller than those usually obtained via measurement of the SVH when subjects are facing forwards [49]. However, responses were not absent in the backward and transverse positions, as appears to be the case in the supine position. Thus, during a simulated co-ordinated turn the perception of tilt with respect to the surface of the Earth is highly dependent on the subject’s orientation with respect to the trajectory.

### 4.2 The SVH-SZ difference in relation to basic properties of the canal system

Mergner et al. [25] studied the perception of yaw angular displacement for several combinations of angular velocity and duration with subjects seated upright. Assuming a time constant for velocity storage of 20 s, their findings suggest that a 60-degree displacement, performed with 5°/s (approximating the swing out of the gondola during acceleration of the centrifuge) would result in a perceived displacement of approximately 40°. Therefore, it might seem remarkable that the responses were virtually zero when this yaw stimulus was encountered in the supine position in the gondola centrifuge.

If focussing on the angular-*displacement* canal component, related to the 60-degree swing out of the gondola during acceleration (i.e. the roll-plane component when the subject is seated upright, the yaw-plane component when he/she is lying supine), the present findings appear to be in conflict with a wealth of data on oculomotor and perceptual responses to rotations in yaw, pitch or roll about a fixed Earth-vertical or Earth-horizontal axis.

Firstly, during oscillation about an Earth-vertical axis the human vestibulo-ocular reflex gain is higher for the yaw plane than for the roll plane [2, 38]. Secondly, the velocity storage function (a short-term memory for ongoing rotation) is more developed for the yaw plane than for the roll and pitch planes. The time constant for decay of per-rotatory canal phenomena is 10–20 s for rotation in yaw, whereas for roll or pitch rotation it is only half of that (for references, see [34, 60]). Thirdly, whereas the persistent otolithic signal for upright position counteracts the canal signal for roll angular displacement when the subject is sitting upright in the gondola [10, 56], there is evidence suggesting that the graviceptive signal would not interfere with the yaw-plane canal message in a supine subject [2]. Thus, Bockisch and co-workers [2] recorded the vestibulo-ocular reflex during angular oscillations in the subject’s yaw, pitch and roll plane and with the axis of rotation either vertical or horizontal. For pitch and roll oscillations responses were greater if the axis of rotation was Earth-horizontal than if it was Earth-vertical; for the yaw plane, in contrast, there was no such effect, presumably because of the lack of otolith contribution. These three properties of the canal system likely represent an adaptation to the conditions of everyday life, where angular displacements in yaw tend to be of greater duration and amplitude but do not necessarily evoke changes in otolith activity.

Obviously, the difference in perceived angular displacement between the upright (gondola displacement in roll) and supine (gondola displacement in yaw) positions cannot be explained by canal system properties revealed in experiments with simple rotations about an Earth-fixed axis. Therefore, in addition to the angular *displacement* component also the angular *velocity* components related to the rotation of the centrifuge about its main axle (i.e. the yaw and pitch components when the subject is upright, the roll and pitch components when the subject is supine) (see Table 1) must be considered. However, neither the pitch nor the yaw component would in itself exert any influence on orientation in the roll plane; pitch rotation is symmetrical with respect to the body, whereas yaw stimuli must be much more intense to induce notable changes in SVH [58].

One possibility is related to the *pattern* of canal stimuli in yaw and pitch. An ideal observer, seated-upright facing-forwards in the gondola, would perceive the initial yaw-left rotation as the entering of a curve, and interpret the increasing sensation of pitch-backward rotation as a consequence of his/her increasing roll tilt with respect to an Earth-fixed axis of rotation. The roll plane component *per se* would facilitate such an interpretation. This means that the perceived plane of rotation (determined by the proportion between the perception of the yaw and pitch angular velocity components) could function as a clue as to the Earth horizontal plane. However, when the subject is sitting upright in the gondola, the difference in velocity storage between yaw and pitch may, according to Melvill Jones et al. [24] cause a tendency for the perceived plane of rotation to approach the subject’s yaw plane, thus impairing the ability to use the perceived pitch component as an indication of being roll tilted with respect to the plane of ongoing rotation. In contrast, if the subject is in the supine position, the velocity storage mechanism (with similar time constants for roll and pitch), would not induce any bias in the perceived gondola inclination.

Consequently, velocity storage functions would rather counteract than contribute to the observed difference between the SVH and SZ responses to acceleration of the centrifuge. Considering the angular-displacement stimulus (the roll component when the subject is upright, the yaw component when he is supine), velocity storage would increase the response when the subject is supine. And considering the pattern of the two angular-velocity components (yaw and pitch when the subject is upright, roll and pitch when he is supine), velocity storage would counteract the response when the subject is sitting upright. In few words, the difference between SVH and SZ cannot be the result of “bottom-up” processing of canal signals, decaying with different time constants for the three planes of rotation.

Nevertheless, in combination the two per-rotatory components (yaw and pitch when the subject is seated upright; roll and pitch when he is supine) reflect a change in head orientation with respect to the plane of centrifuge acceleration. The relative intensity of these components is a function of the swing out of the gondola. To a subject who is seated upright in the gondola, the yaw-plane component predominates in the beginning of acceleration, but when the inclination of the gondola exceeds 45 degrees the pitch-backward component will be greater (Fig. 1, Table 1). If the vestibular system were capable of “tracking” the axis (or plane) of rotation, taking it for an Earth-fixed reference, then the yaw and pitch components would contribute to the sensation of change in *roll* position. This cannot, however, explain the forward-facing/backward-facing asymmetry in SVH [47] unless we assume that also the *familiarity* of a specific pattern of yaw and pitch stimuli is an essential factor. Namely, the pattern experienced in the forward position would be more familiar than that in the backward or supine position.

### 4.3 Top-down processing

A sensory system is often characterised as multiple parallel streams, each with a hierarchy of mechanisms from the receptor organs to the level of conscious experience. Because of bidirectional connections, processing at lower levels is not entirely determined by input from the periphery. Even in the primary visual cortex the responsive properties of neurons or neuronal circuits are sensitive to context [32] and they can be modulated by top-down factors, such as familiarity, expectations and attention [9, 31]. The prefrontal cortex is likely to play a significant role by facilitation of appropriate perceptual processes at lower levels [43].

As noted by Holly and McCollum [15] computational models for human self-motion perception are usually dominated by “bottom-up” principles, and perceived motion is obtained as a function of the various inputs from receptors. This approach has, indeed, successfully explained several vestibular phenomena studied in classical experimental set-ups [15]. Nevertheless, even in static conditions, measures of spatial orientation may be influenced via top-down processing. Mast and co-workers [22] found that the mere imagination of tilted gratings induced deviations in the SVV. Extending these observations, Mertz and Lepecq [26] showed that imagined body tilt causes deviations in the SVV similar to those recorded during physical body tilt. A model for the case of head rotation has been developed by Jürgens and Becker [17], demonstrating that perceived angular displacement is compatible with a mechanism using probabilistic fusion in two steps. First, a weighted average of “bottom-up” information (e.g. visual and canal input) is generated; the second step consists in fusion of this combined sensory input with “top-down” information (e.g. expectations). The weight of top-down information would be greater in case sensory information is scarce or noisy [17]. More generally, the significance of learning and top-down modulation seems likely to increase with the complexity of movement patterns.

### 4.4 Gestalt psychological mechanisms in pilot learning and vestibular perception

According to Gestalt psychology, the whole is more than the sum of its parts. Even before Werthei-mer (inspired by illusory motion perception) published his pioneering work in 1912 [59], the Czech philosopher and psychologist von Ehrenfels had emphasised that many complex phenomena, like melodies and works of architecture, possess Gestalt qualities, the perception of which appears to be independent of conscious recognition of details [57]. By creating overall impressions of objects or events, the perceptual process reduces the load of information on the conscious mind and working memory [20, 36, 45]. The “laws” of Gestalt psychology state that the grouping of elementary sensory data tends to generate perceptions that are simple, harmonious or have few parts [11].

Another central idea within Gestalt psychology is that perception to a significant extent is determined by expectations and a readiness to recognise familiar patterns [3]. A general effect of experience is the formation of associations. Larger networks of associated memories imply a higher likelihood that a single cue will evoke whole chunks of information. Such networks are not unstructured. Rather, practice generates mental schemata, which are tendencies to perceive information in particular ways and to recognize patterns. Experiments on chess players have revealed that expertise dramatically improves the capacity to memorize complex constellations of pieces - if these constellations are logically possible. When it comes to random configurations, however, expert players are not better than novices [4]. The ability to recognize patterns also constitutes a link between perception and action. Notably, the Gestalt psychological idea of complex entities processed as single units has been applied also to motor responses [19]. This is of particular interest as regards the acquisition of skills involving self motion.

Learning in pilots, and their ability to integrate rather artificial pieces of visual information into meaningful wholes, as well as generating coordinated and fine-tuned responses with little intellectual effort, might serve as an illustration of the mechanisms and potential of higher level perceptual organi-zation and motor control. Since the movements of an aircraft cannot be adequately perceived via the sense of balance [33] it is essential, in conditions of poor visibility, that the pilot ignores vestibular impressions and bases the manoeuvring on scanning and interpretation of cockpit instruments. With flight training there is an increasing tendency to perceive the indications by separate instruments as chunks. Similarly, responses with the control column, pedals and throttle tend to be performed as units. As an analogy to the above-mentioned findings on chess players, the capability to reconstruct snapshots of simulated cockpit situations (including instrument indications) was better in experienced pilots than in novices. However, if the presented situations were not meaningful or coherent with real scenarios, the performance of expert pilots did not differ from that of novices [40]. These results suggest that practice strengthens a certain functional relationship between working memory and long-term memory, enabling use of sophisticated knowledge via few and simple elements controlled by the intellect.

Evidence to the fragility of these mechanisms is also provided by aviation. During acute psychological stress the release of dopamine and noradrenalin in the prefrontal cortex may induce a switch in the control of behaviour from the top-down cognitive domain to the bottom-up level characterized by more direct or instinctive responses to sensory impressions [1]. In addition, severe stress causes a restriction of the field of attention, such that even the experienced pilot may focus entirely on one single instrument [37]. Such coning of attention, as well as the tendency to manoeuvre the aircraft guided by vestibular illusions, is an important cause of accidents [52]. The study of vestibular mechanisms for self-motion perception has been motivated to a great extent by this spatial disorientation problem. From that point of view, formal modelling might facilitate the creation of adequate stimulus patterns in flight simulators.

Holly and McCollum [15] refer to several experiments with complex vestibular stimuli where the perceptual outcome is difficult to explain without principles of Gestalt psychology. In one notable study, Guedry and co-workers [13] asked test subjects to carefully describe their experiences of self-motion during acceleration and deceleration in a gondola centrifuge. Subjects were tested in four different positions, facing forwards, backwards and centripetally as well as in the supine position with the feet towards the centre of the centrifuge. A general finding was that the perceived motion patterns were not concordant with recordings of vestibulo-ocular reflexes and could not be explained by existing models for vestibular perception [13]. According to Holly and McCollum [15], certain complex movements would be perceived as meaningful wholes, in analogy with visual objects or spoken words. Although the repertoire of “pattern detectors” or “mental schemata” is likely to be smaller for the sense of balance than for vision and audition, several parallels between vestibular perception and other sensory modalities have been identified [15]. In the “whole-motion model” [14] principles of experience and familiarity are applied to the stimulus pattern as a whole in three dimensions. The model [14] provides an explanation for the forward-facing/backward-facing difference in perceived roll tilt during gondola centrifugation [47]. In contrast, the parameters of a “Component-wise Model”, where the concept of familiarity is applied at the level of simple stimulus components, could not be adjusted to match the observed difference in perceived roll tilt [14].

In the present study the stimulus created with the subject in the forward position, which may be considered a “natural” stimulus pattern, was contrasted with a position where the angular displacement of the gondola coincides with the subject’s yaw plane, i.e. the plane where our canal system is most responsive. Nevertheless, the measure of perceived angular displacement was greater in the upright position. This indicates that a subject seated upright facing forwards perceives the angular displacement of the gondola not as an isolated stimulus component; rather, its perception is dependent on the recognition of a complex pattern related to a specific situation, namely the entering of a coordinated turn. In other words, a component (e.g. roll angular displacement) would more likely be detected or correctly estimated if the context or situation (e.g. the entering of a turn) has been adequately recognized. This seems to be another analogue of the capability of expert chess players to recall the details of meaningful configurations of pieces. Hypothetically, if the perception of a single stimulus component (e.g. the roll angular displacement of the gondola) is dependent on the subject’s ability to identify the complex motion pattern as a meaningful whole, then this would also suggest a possibility of studying the recognition of complex motion patterns by recording a single perceptual component.

### 4.5 The entering of a co-ordinated turn

Individuals who often experience coordinated turns, for instance aviators, show greater and more lasting SVH tilts during centrifugation [53], suggesting that the ability to identify this motion pattern may be acquired via vestibular learning. Further, recordings of the SVH in the gondola centrifuge and an aircraft suggest that the stimulus situation created in the centrifuge is perceptually similar to a real coordinated turn [54]. Nevertheless, because of the difference in radius between the aircraft turn and the centrifuge, in the aircraft the values for the yaw and pitch components were only about 10% of their values in the centrifuge. This means that the forward-facing/backward-facing asymmetry in perceived roll tilt during centrifugation [47] is not necessarily the result of a *contribution* of the yaw and pitch components (i.e. in addition to that induced by the roll stimulus) when the subject is facing forwards; if that were the case, the SVH tilt would have been smaller in the aircraft than in the centrifuge. Rather, when the subject is facing backwards in the centrifuge the pattern of yaw and pitch stimuli is likely to be *unfamiliar*, and this would pertain also to the stimulus pattern as a whole. An alternative interpretation would be that in the backward position the unfamiliar pattern of the yaw and pitch components (which are of greater magnitude than the roll component) is distracting, and therefore interfere with the perception of the roll angular displacement. But even that interpretation relies on the concept of familiarity.

The stimuli and findings of the present study may be pondered upon in an analogous way. The question is whether the SVH tilt obtained in the forward position is a result of the brain’s recognition of a meaningful stimulus pattern or whether the absence of deflection in SZ is due to the fact that the motion pattern is most unfamiliar - and that the unfamiliarity prevents the formation also of a percept of the simple yaw plane component. In this connection it should not be forgotten that also in the forward position the underestimation of the gondola’s angular displacement is usually considerable. In real life, the vestibular stimulus pattern of entering a co-ordinated turn is associated with visual impressions, motor activity and intentions. Thus, as generated in the centrifuge the stimulus pattern may lack important qualities even if the subject is in the upright and forward-facing position.

### 4.6 The significance of early cues

Situational awareness and early cues influence perception by evoking expectations. This would be inconceivable without preconceptions of stimulus patterns. Familiarity or meaning of a stimulus implies that the brain has a certain readiness to detect it, and this may be called ‘pattern detector’ or ‘Gestalt’. Our tendency to respond to patterns, e.g. words, can be increased by early and subtle cues, even if these do not reach conscious level, a phenomenon called priming [7, 41]. Thus, an early cue, which might as well consist in background conditions or - as demonstrated by Pavlov [35] - a signal whose nature is very different from the main stimulus, enhances the responsiveness of the pattern detector. Further, if the subject is thereby *expecting* the following pattern he or she would also be more apt to discern its components or to estimate their magnitudes. As vestibular stimuli and responses can be characterized in quantitative terms, this field might afford opportunities to formally study how various factors initiate, or modify, top-down processing.

According to the whole-motion model a stimulus component that predominates in the beginning of a complex pattern can determine the overall perceptual effects [14]. In the present experiments, two early cues are the tangential jerk and the angular acceleration of the centrifuge about its main axle, i.e. yaw left (subject sitting) or roll right (subject supine). With an angular acceleration of the centrifuge of 7°/s^2^, this angular velocity component will be well above the stimulus threshold of the canals within 1s. As humans are more keen to perceive rotations or angular displacements in yaw than in roll, this kind of early cue would have a greater impact when the subject is upright than when supine. An upright subject, who senses the initial yaw-left rotation, will experience the subsequent pattern as the entering of a left-turn *combined* with a leftward roll tilt. In contrast, when the subject is in the supine position the initial roll-right stimulus is less tangible; and if there is no sensation of entering a curve in the Earth-horizontal plane, then neither would the yaw movement be sensed. Rather, the increasing pitch-backward angular-velocity component, combined with the tangential acceleration, may induce an illusion of movement along a trajectory deviating upwards.

## 5 Conclusion

The present findings complement those of earlier centrifuge experiments on how the subject’s heading position determines the ability to perceive the gondola’s angular displacement during acceleration. In the present study, the “natural” forward-facing position was contrasted with the supine position. When the subject is supine the angular displacement of the gondola coincides with the yaw plane, i.e. the plane for which the canal system is most responsive. Nevertheless, in that condition the visual measure of perceived angular displacement was virtually zero. This is difficult to explain in a bottom-up manner from stimulus components, but lends support to theories on Gestalt psychological mechanisms in vestibular perception, according to which the ability to perceive the displacement of the gondola is dependent on the familiarity of the stimulus pattern as a whole in 3D. As experienced in the upright forward-facing position the pattern would be more familiar, promoting also the subject’s ability to discern and estimate a single stimulus component. More generally, during complex vestibular stimulation the ability to perceive a single component might reflect the degree of familiarity of the pattern as a whole.
